# Childhood stunting in Thailand: when prolonged breastfeeding interacts with household poverty

**DOI:** 10.1186/s12887-018-1375-5

**Published:** 2018-12-27

**Authors:** Nisachol Cetthakrikul, Chompoonut Topothai, Rapeepong Suphanchaimat, Kanjana Tisayaticom, Supon Limwattananon, Viroj Tangcharoensathien

**Affiliations:** 1 International Health Policy Program, Ministry of Public Health, Nonthaburi, Thailand; 2 Department of Health, Ministry of Public Health, Nonthaburi, Thailand; 3 Bureau of Epidemiology, Department of Disease Control, Ministry of Public Health, Nonthaburi, Thailand; 4 Faculty of Pharmaceutical Sciences, Khon Kaen University, Khon Kaen, Thailand

**Keywords:** Stunting, Children, Breastfeeding, Thailand, Household-wealth

## Abstract

**Background:**

Childhood stunting, defined as the height-for-age standardized score lower than minus two, is one of the key indicators for assessing well-being and health of a child; and can be used for monitoring child health inequalities. Thailand has been successful in improving health and providing financial protection for its population. A better understanding of the determinants of stunting will help fill both knowledge and policy gaps which promote children’s health and well-being. This study assesses the factors contributing to stunting among Thai children aged less than five years.

**Methods:**

This study obtained data from the Multiple Indicator Cluster Survey Round 4 (MICS4), conducted in Thailand in 2012. Data analysis consisted of three steps. First, descriptive statistics provided an overview of data. Second, a Chi-square test determined the association between each covariate and stunting. Finally, multivariable logistic regression assessed the likelihood of stunting from all independent variables. Interaction effects between breastfeeding and household economy were added in the multivariable logistic regression.

**Results:**

In the analysis without interaction effects, while the perceived size of children at birth as ‘small’ were positively associated with stunting, children in the well-off households were less likely to experience stunting. The analysis of the interactions between ‘duration of breastfeeding’ and ‘household’s economic level’ found that the odds of stunting in children who were breastfed longer than 12 months in the poorest household quintile were 1.8 fold (95% Confidence interval: 1.3–2.6) higher than the odds found in mothers from the same poorest quintiles, but without prolonged breastfeeding. However prolonged breastfeeding in most well-off households (those between the second quintile and the fifth wealth quintile) did not show a tendency towards stunting.

**Conclusions:**

Childhood stunting was significantly associated with several factors. Prolonged breastfeeding beyond 12 months when interacting with poor economic status of a household potentiated stunting. Children living in the least well-off households were more prone to stunting than others. We recommend that the MICS survey questionnaire be amended to capture details on quantity, quality and practices of supplementary feeding. Multi-sectoral nutrition policies targeting poor households are required to address stunting challenges.

## Background

Maternal and child health (MCH) has been globally recognized as one of the key indicators in measuring health system performance. The Millennium Development Goals (MDG), set the target to reduce under-five mortality rate and maternal mortality rate by two thirds and three quarters respectively by 2015 [1, 2]. To rectify the unfinished MCH goals in the MDG era, the Sustainable Development Goals (SDG)-3, established the ambitious targets of global maternal mortality reduction to less than 70 deaths per 100,000 live births, and to ending preventable deaths of children under 5 years of age by 2030 [3]. The MCH goal was also linked with the SDG-2 where the United Nations (UN) Member States committed to promote food security and improve the nutritional status of vulnerable populations, including children under 5 years of age, adolescent girls, pregnant and lactating women, and the elderly [4].

The World Health Organisation (WHO) proposed 11 indicators for MCH monitoring—maternal mortality, prevalence of stunted children, exclusive breastfeeding for 6 months after birth, and skilled birth attendants, to name a few [5]. Amongst these indicators, De Onis and Branca suggest that childhood stunting is ‘the best overall indicator of children’s well-being and an accurate reflection of social inequalities’ [6]. The WHO also highlighted that stunting is a result of long-term malnutrition, poor diets and nutritional deprivation—leading to growth retardation, delayed mental development, poor school performance and reduced intellectual capacity [7, 8]. The consequences of stunting are catastrophic, not only in terms of health but also in terms of economic outcomes. The World Bank suggested that a 1% increase in loss of height is associated with a 1.4% loss in economic productivity [9].

The global prevalence of stunting is extremely high. Around 155 million children under 5 years of age are experiencing stunting [10]. The situation is most severe in Africa and Southeast Asia, which account for about 34% of the global stunting prevalence [11].

A number of factors contribute to stunting: political economy; health and health care systems; education, society and culture; agriculture and food systems; and water, sanitation and environment [12]. Poor hygiene and low breastfeeding rates were associated with stunting [13], and low birth weight and prolonged breastfeeding (more than 12 months) without adequate and appropriate supplementary feeding, and diarrhoea were among major risk factors [14].

Factors that contribute to stunting can be categorized as (1) child conditions, such as non-exclusive breastfeeding and discontinuity of breastfeeding before 2 years, slow fetal growth during pre-natal period, and having been born prematurely [9, 15]; (2) maternal conditions, including poor nutrition and health status of a mother during pre-natal, peri-natal, and post-natal phases, and inadequate preparation of a mother during pregnancy [9]; and (3) socio-economic factors, both macro-level determinants (lack of sanitation and an unhealthy environment for child rearing), and micro-level determinants (household poverty, improper childrearing practices, and poor maternal education) [14].

Thailand has been successful in improving health outcomes and providing financial protection for its population in spite of relatively low healthcare spending [16]. The infant mortality rate was markedly reduced from 26 in 1990 to 11 per 1000 live births in 2010 [17]. Despite this, attempts to combat childhood stunting are not progressing well. Although the early 1990s marked a success in reducing stunting prevalence from over 22% in 1987 to around 12% in 1995, the stunting trend has stagnated and never fallen below 10% since then [13]. Child stunting was found to be the second most concentrated MCH problem amongst the poor, following only the problem of underweight children [18]. Moderate and severe stunting stood at 10.5% in 2016. The prevalence amongst households headed by a non-Thai speaker was more pronounced at 16.3% [19]. The path to tackling the problem of stunting is not paved with roses, as a case study from Thailand clearly reflects. In response to the repeated violations of the Code of Practice on Marketing of Breast Milk Substitutes by milk-substitute industries [20], in 2017 the Thai Ministry of Health (MOPH) introduced a draft Bill to regulate such interferences by the industries. However, there was strong resistance from certain paediatricians and the Medical Council who amplified the downsides of prolonged breastfeeding.

Though several studies on childhood stunting describe the magnitude of stunting, comprehensive assessment of its social determinants is lacking [21, 22]. A better understanding of contributing determinants will help policy makers to devise proper comprehensive multi-sectoral policies to alleviate stunting prevalence. Therefore, this study aimed to assess potential factors contributing to stunting amongst Thai children below 5 years of age, in particular those who were breastfed beyond 12 months.

## Methods

### Operational definition

Prevalence of stunting is defined as the percentage of children under age five whose height for age is greater than two-fold of standard deviations at the direction below the international reference median for children aged 0–59 months. Note that for children up to two years old, height is measured by recumbent length unlike older children where height is measured by stature while standing [23].

### Study design and data source

This study applied a cross sectional quantitative design. Data were obtained from the Multiple Indicators Cluster Survey (MICS) in 2012, which was jointly conducted by the International Health Policy Program (IHPP), the MOPH, the National Statistical Office (NSO), the Thai Health Promotion Foundation and the United Nations Children’s Fund (UNICEF) in 2012. Multi-stage stratified cluster sampling was applied. Enumeration areas (EAs) and households served as primary and secondary sampling units respectively (approximately 20 households in each EA). The overall sampling frame is presented in Table 1.

**Table 1:** Number of sample household and response rate of the MICS round 4, Thailand

Region	No. of household		Response rate
	Samples	Interviewed	
Bangkok	2959	2480	95.8
Central	5970	5190	98.6
Northern	5945	5532	99.5
Northeastern	5999	5631	99.6
Southern	5977	5286	97.7
Total	26,850	24,119	98.5

A total of 24,119 households participated in the survey from a target of 26,850 (response rate = 98.5%). Amongst these households, 9716 children were recruited. As this study focused on factors that significantly contribute to stunting, particularly the effects of prolonged breastfeeding, we limited our analysis only to children aged over 12 months (as prolonged breastfeeding applied the cut-off at 12 months), numbering 7018 in total.

### Data collection, questionnaire design, and variable management

Face-to-face interviews with mothers and/or legal guardians were performed in each household by the NSO field staff. Each interview took an average of 60 min to complete. Field staff entered data directly into the mobile tablets. Revisiting was done if mothers at the visited household were absent during the first round of survey.

The standard MICS questionnaire was used [24]. All variables were those collected earlier in the prior rounds of MICS [9, 14, 15]—for instance, history of breastfeeding; maternal education; and location of a household, and also other variables such as geographic region; housing characteristics; economic status; maternal antenatal care history; and health status of a child before the survey period.

The variables of our focus were (1) current age (months), height (cm), and weight (kg) of a child for measurement of moderate and severe stunting; (2) pregnancy age at delivery (less than 20 years, 20–29 years, 30–39 years, and equal to or more than 40 weeks); (3) maternal perception on the size of her baby at birth (small, average, large, and ‘cannot clearly determine the size’); (4) history of diarrhoea during the last two weeks before the survey (yes v no); (5) educational background of mother (no education, primary school completion, secondary school completion, diploma, bachelor degree, and above bachelor degree); (6) household domicile (municipality [representing urban] v outside municipality [representing rural]); (7) geographic region of the household (Bangkok, Central, Northern, Northeastern, and Southern); (8) antenatal care (ANC) history (4 visits or more, fewer than 4 visits, and ‘cannot remember visit number’); (9) breastfeeding history (less than 12 months v 12 months or more), and; (10) household asset index (poorest, poor, moderate, rich, and very rich asset quintiles). Note that the World Bank recommends using asset index as the indicator for measuring household’s economy over monetary income [25]. The index was calculated by principal component analysis, taking into account all important durables, land possession, household infrastructure, and domestic commodities.

### Data analysis

We used STATA software version 14 (serial license number: 401406358220) to analyse the data. The analysis was divided into three steps. Firstly, descriptive statistics were done to provide the overview of the data. Secondly, a univariable analysis by Chi-square test was done to determine the association between each covariate and stunting. Lastly, multivariable logistic regression was applied to assess the likelihood of stunting after accounting for the effects of all covariates at the same time. All covariates from the univariable analysis were included in the multivariable analysis regardless of the significance level, since we hypothesized that these variables were likely related to stunting, as supported by much literature in the introduction section.

As stunting can be a result of complex interactions between various social determinants, we then divided the multivariable analysis into two strands: first, the analysis without interaction terms and second, the analysis with interaction terms. In this regard, a new variable representing the interactions between breastfeeding duration and a household’s socio-economic profile was included in the final model. Robust standard error was used to adjust for potential cluster effects within a household. Crude and adjusted odds ratios (OR) with 95% confidence interval (95% CI) were presented. Statistical significance was measured at the 95% confidence level (*P*-value < 0.05).

## Results

### Descriptive statistics and sample characteristics

A total of 7018 children under 5 years old were enrolled in this study. Around three quarters of them were born from mothers who had completed at least high-school education and gave birth at age between 20 and 39 years. The majority of those mothers for whom ANC information was available had adequate ANC visits. About 88.74% of the children were not currently breastfed. Only 30.8% of the children had been breastfed for more than 12 months. Most mothers (71.0%) did not have adequate information about the size of their child at birth. However, for those mothers who could determine the size of their child at birth, the most common report was ‘average’ (21.4%), followed by ‘large’ (4.9%). Over 95.7% of the children did not have recent experiences of diarrhoea and approximately 14.1% of the children experienced stunting.

Regarding household attributes, around three quarters were ranked in the 3rd to 5th quintiles of the wealth index. Those located in a municipal area slightly outnumbered those living in rural areas. The samples were quite equally shared between the different geographic zones except Bangkok (Central 21.0%, Northern 22.0%, Northeastern 23.5%, Southern 24.2% and Bangkok 9.3%) as depicted in Table 2.

**Table 2:** Key characteristics of the participants*

Factors	No (%)
Parental characteristics	
Maternal education	
No education	211 (3.0)
Primary	1973 (28.3)
Secondary	2709 (38.8)
Diploma	791 (11.3)
Bachelor’s degree	1201 (17.2)
Master’s degree and above	97 (1.4)
Maternal age at birth	
< 20 years	672 (9.8)
20–29 years	2996 (43.9)
30–39 years	2456 (36.0)
40 years and above	701 (10.3)
Number of ANC visits	
< 4 Times	65 (0.9)
≥ 4 Times	1892 (27.0)
Cannot remember visit number	5061 (72.1)
Currently breastfeeding	
Yes	750 (11.3)
No	5887 (88.7)
Duration of breastfeeding	
≤ 12 months	4591 (69.2)
> 12 months	2043 (30.8)
Child characteristics	
Perceived size of baby at birth	
Small	188 (2.7)
Average	1503 (21.4)
Large	341 (4.9)
Cannot clearly determine the size	4986 (71.0)
Child’s age (months)	
13–24 months	1696 (24.3)
25–36 months	1836 (26.3)
37–48 months	1791 (25.6)
49–59 months	1662 (23.8)
History of recent diarrhoea	
Yes	302 (4.3)
No	6670 (95.7)
Stunting	
No	5626 (85.9)
Yes	925 (14.1)
Household level factors	
Wealth index	
Poorest	914 (13.1)
Poorer	1182 (16.9)
Middle	1537 (22.0)
Richer	1735 (24.9)
Richest	1610 (23.1)
Community level factors	
Type of residence	
Municipal	3695 (52.7)
Non-municipal	3323 (47.3)
Geographic zone	
Bangkok	654 (9.3)
Central	1474 (21.0)
Northern	1543 (22.0)
Northeastern	1650 (23.5)
Southern	1697 (24.2)

Note:

• As the volume of missing data in each variable was small, missing data were not reported in the table

• The wealth index variable was analyzed by the NSO based on the full samples, while this study focused on household of over-12-month children. Thus, the proportion of our samples for each quintile was not exactly equal to 20%

### Univariable analysis

The results of univariable analysis are described in Table 3. Clearly, a high maternal education background and increased age at delivery had an inverse relationship with the probability of childhood stunting. Prolonged breastfeeding was positively associated with stunting (OR = 1.57, 95% CI 1.35–1.81). Stunted children tended to be the younger age groups (less than 36 months), and were those who had a small size at birth, as reported by their mothers. Furthermore, the finding shows that children brought up in the better-off households, and those living within municipalities, had lower stunting probability compared to those from poor socio-economic backgrounds. Note that geographic residence was not significantly associated with stunting.

**Table 3:** Univariable analysis on stunting

Factors	Odds ratio (SE)	95% CI
I. Parental level factors		
Maternal education (v no education)		
Primary	0.46 (0.08)	0.33–0.64***
Secondary	0.37 (0.06)	0.27–0.51***
Diploma	0.30 (0.06)	0.21–0.44***
Bachelor’s degree	0.26 (0.05)	0.18–0.37***
Master’s degree and above	0.41 (0.14)	0.22–0.79*
Mother’s age at birth (v < 20 years)		
20–29 years	0.84 (0.10)	0.66–1.05
30–39 years	0.66 (0.08)	0.52–0.84***
> 40 years	0.68 (0.11)	0.50–0.93*
Number of ANC (v < 4 times)		
≥ 4 Times	0.64 (0.20)	0.35–1.18
Cannot remember visit number	0.48 (0.15)	0.26–0.89*
Duration of breastfeeding (v < 12 months)		
> 12 months	1.57 (0.12)	1.35–1.81***
II. Child level factors		
Perceived size of baby at birth (v small)		
Average	0.72 (0.14)	0.49–1.06
Large	0.59 (0.14)	0.36–0.95*
Cannot clearly determine the size	0.52 (0.10)	0.36–0.76***
Child age in months (v 12–23 months)		
24–35 months	0.83 (0.08)	0.69–1.00
36–47 months	0.75 (0.07)	0.62–0.90***
48–59 months	0.61 (0.06)	0.50–0.75***
History of recent diarrhoea (v Yes)		
No	0.92 (0.16)	0.66–1.28
III. Household level factors		
Wealth index (v poorest)		
Poorer	0.69 (0.08)	0.55–0.87***
Middle	0.59 (0.06)	0.47–0.73***
Richer	0.46 (0.05)	0.37–0.58***
Richest	0.39 (0.05)	0.31–0.49***
IV. Community level factors		
Type of residence (v municipal)		
Non-municipal	1.25 (0.09)	1.09–1.44***
Geographic zone (v Bangkok)		
Central	1.00 (0.15)	0.74–1.34
Northern	0.94 (0.14)	0.70–1.26
Northeastern	1.02 (0.15)	0.76–1.36
Southern	1.22 (0.18)	0.92–1.63

Note: * P-value < 0.05, **P-value < 0.01, and *** P-value < 0.001

### Multivariable analysis

Table 4 presents the outcomes of multivariable analysis (both with and without interaction effects). In the analysis with no interaction effects, higher maternal education and the more advanced the age of mothers at the time of delivery lowered the probability of stunting. The perceived size of children at birth as ‘average’ or ‘large’ yielded smaller odds of stunting compared to those with a low birth weight who were reported by mothers as ‘small’ in size. Children in the better-off households were less likely to face stunting. Prolonged breastfeeding was positively associated with stunting. Other covariates, such as number of ANC visits and history of diarrhoea within the last two months, appeared to have insignificant effects on stunting.

**Table 4:** Multivariable analysis on stunting with and without interaction effects between duration of breastfeeding and wealth index

Factors	With interaction terms		Without interaction terms	
	Odds ratio (SE)	95%CI	Odds ratio (SE)	95%CI
I. Parental level factors				
Maternal education				
No education	1.00		1.00	
Primary	0.54 (0.12)	0.35–0.82***	0.55 (0.12)	0.36–0.84*
Secondary	0.42 (0.09)	0.27–0.64***	0.42 (0.09)	0.28–0.65***
Diploma	0.40 (0.10)	0.25–0.64***	0.40 (0.10)	0.25–0.65***
Bachelor’s degree	0.41 (0.10)	0.25–0.66***	0.41 (0.10)	0.26–0.67***
Higher Bachelor’s degree	0.76 (0.29)	0.37–1.59	0.77 (0.29)	0.37–1.60
Mother’s age at birth				
< 20 year	1.00		1.00	
20–29 years	0.89 (0.11)	0.69–1.13	0.89 (0.11)	0.70–1.13
30–39 years	0.68 (0.09)	0.52–0.89***	0.68 (0.09)	0.52–0.89*
40 years and above	0.60 (0.11)	0.42–0.85*	0.60 (0.11)	0.42–0.86*
Duration of breastfeeding				
< 12 months	1.00			
> 12 months	1.35 (0.11)	1.15–1.58***		
Number of ANC*				
< 4 Times	1.00		1.00	
≥ 4 Times	1.03 (0.35)	0.53–2.01	1.02 (0.35)	0.52–2.00
Cannot remember visit number	1.42 (0.66)	0.57–3.51	1.42 (0.66)	0.57–3.52
II. Child level factors				
Perceived size of baby at birth*				
Small	1.00		1.00	
Average	0.68 (0.14)	0.45–1.03	0.69 (0.15)	0.45–1.04
Large	0.59 (0.15)	0.36–0.98*	0.59 (0.15)	0.35–0.97*
Cannot clearly determine the size	0.38 (0.14)	0.19–0.78*	0.38 (0.14)	0.18–0.78*
History of recent diarrhoea				
Yes	1.00		1.00	
No	1.07 (0.21)	0.73–1.56	1.07 (0.21)	0.73–1.56
III. Household level factors				
Wealth index				
Poorest	1.00			
Poorer	0.74 (0.09)	0.58–0.95*		
Middle	0.60 (0.08)	0.47–0.77***		
Richer	0.48 (0.07)	0.37–0.63***		
Richest	0.46 (0.07)	0.33–0.63***		
IV. Community level factors				
Type of residence				
Municipal	1.00		1.00	
Non-municipal	1.13 (0.09)	0.96–1.32	1.12 (0.09)	0.96–1.32
Geographic zone				
Bangkok	1.00		1.00	
Central	0.76 (0.12)	0.56–1.05	0.77 (0.13)	0.56–1.06
Northern	0.59 (0.10)	0.43–0.82***	0.59 (0.10)	0.43–0.82***
Northeastern	0.60 (0.10)	0.44–0.83***	0.60 (0.10)	0.43–0.83***
Southern	0.90 (0.14)	0.66–1.23	0.91 (0.14)	0.67–1.24
V. Duration of breastfeeding and Wealth index				
BF < 12 months and Poorest			1.00	
BF < 12 months and Poor			0.86 (0.15)	0.61–1.21
BF < 12 months and Middle			0.67 (0.12)	0.48–0.94*
BF < 12 months and Rich			0.65 (0.11)	0.46–0.92*
BF < 12 months and Richest			0.50 (0.10)	0.34–0.74***
BF > 12 months and Poorest			1.79 (0.32)	1.26–2.55***
BF > 12 months and Poor			1.16 (0.22)	0.80–1.67
BF > 12 months and Middle			0.98 (0.19)	0.67–1.44
BF > 12 months and Rich			0.53 (0.11)	0.35–0.81***
BF > 12 months and Richest			0.80 (0.19)	0.51–1.27

Note: * *P*-value < 0.05, **P-value < 0.01, and *** *P*-value < 0.001

The interactions between the duration of breastfeeding and household economic levels contributed to stunting with varying degrees of statistical significance. By using the interaction between ‘poorest wealth index’ and ‘duration of breastfeeding of less than 12 months’ as a reference, it appears that wealth provided a protective effect against stunting regardless of the duration of breastfeeding; but amongst mothers in the poorest quintiles, beyond-12-months breastfeeding tended to contribute to stunting relative to those with shorter duration of breastfeeding. The direction of odds ratio and significance level of other covariates in the analysis with interaction terms did not show a remarkable difference from the analysis without interaction terms.

## Discussion

This study sheds light on risk factors of stunting amongst Thai children under-five years of age through the analysis of the 2012 MICS data. The household’s economic status was the strongest influence on stunting. Children in the poorest quintile were about two-fold more likely to experience stunting than those in the richest quintile.

The discovery from this study supports findings from some prior research. For instance, the UNICEF data and the Global Nutrition Report suggested that stunting prevalence amongst children in the poorest households was more than double those in the richest households [10, 26]. Furthermore, studies in Burundi and Nepal found that economically poorer households had a greater association with stunting and severe stunting than the richer ones [27, 28]. Likewise, in Nigeria, low household wealth index was a risk factor for severe stunting [29].

However, when it comes to breastfeeding, the findings should be interpreted with caution. While Tiwari et al. from Nepal [28] and Akombi et al. from Nigeria [29] reported that a long duration of breastfeeding contribute to stunting, our study found that prolonged duration of breastfeeding by itself is not a strong determinant for stunting, but rather, the interaction between breastfeeding duration and wealth status. Also, this point is largely overlooked in most existing literature on this subject. Children with beyond-12-months breastfeeding in the poorest households were most prone to stunting while children in the well-off households were less likely to suffer from stunting regardless of their breastfeeding duration. Theoretically, long-term breastfeeding creates substantial benefit to the health of mothers and children. Previous studies [27, 30–32] found that continued breastfeeding positively affected children’s development – reducing the risk of autism spectrum disorder (ASD) and increasing cognitive development. In addition, mothers who breastfeed have a lower risk of chronic diseases [32].

A potential explanation for this phenomenon is that the poorest families might not have means to sufficiently provide an adequate amount of appropriate complementary food for their children after the first six-months of exclusive breast feeding. As a result, breastfeeding was continued as the only viable choice, but its nutrient content alone is not sufficient to match the increased demand of children as they grow up. This coincides with the WHO’s recommendation, suggesting that exclusive breastfeeding should be performed for the first 6 months. After that, complementary food should be provided in addition to breastfeeding. Unfortunately, the current version of the MICS questionnaire does not allow for detailed analysis of complementary food acquisition.

To unpack this complexity, UNICEF should, in the future, amend the MICS survey questionnaire to reflect the magnitude and profile of supplementary food feeding and feeding management. Additionally, some literature mentions the effects of the practice of six-month exclusive breastfeeding as a factor that contributes to optimal child growth [33, 34]. This may also explain the stunting phenomenon in this study as the exclusive breastfeeding rate of Thai infants during the first six months of life was consistently low (MICS 3 in 2009 and MICS4 in 2012 show that the exclusive breastfeeding rate was only 5.4% [35] and 12.3% respectively) [24].

For other covariates, those with low birth weight or who were born premature were about 30–40% more likely to develop stunting than those with a normal or large body size at birth. Surprisingly, children residing in more affluent areas, like Bangkok, were more likely to face stunting than those in other regions. This finding is in contrast to some of the international literature on this subject which has found childhood stunting to be more prevalent in rural than urban households [6, 26, 36]. The child-rearing behaviour of mothers in Bangkok, who were mostly employed in the service and manufacturing sectors and as a result had limited time, which subsequently affected the quality of child rearing, and the nuclear-family characteristic of most households in Bangkok where little or no support from relatives is provided to mothers, are possible explanations.

Despite a rigorous sampling technique and a large number of participants, which are the strengths of the study, certain limitations remain. Firstly, the questionnaire did not contain questions on child rearing practices, especially on complementary food provision in terms of both quality and quantity. Poverty, lower maternal education and lack of adequate complementary food amongst the poorest households can be significant risk factors for stunting. Without this information, the association between duration of breastfeeding beyond 12 months and stunting can be misinterpreted, and send the ‘wrong signal’ to the society about the downside of breastfeeding. It can be used wrongly by the ‘pro-breast milk substitutes’ advocates to refute the benefits of breastfeeding. In fact, there was a question about food security in the MICS questionnaire, that is, ‘Did your child have the following food in the last 24 hours?’. The respondents were asked to answer ‘yes’ or ‘no’ for each food item on the list, for instance, water, condensed milk, rice, and fruit. However, it seems that the question was not well crafted, making it difficult to serve as an indicator for complementary food consumption. Therefore, it was dropped from our analysis.

Secondly, the sampling process relied on the household registry from the Department of Provincial Administration. Although this is a normal process, used by other NSO surveys, there is a downside to this approach. That is, people whose households do not have a registry number, especially disadvantaged populations such as undocumented migrants, homeless people, and slum dwellers, were likely to be excluded from the sampling frame. As a result, the results might underestimate stunting prevalence as the analysis may be prone to missing the poor households.

Thirdly, the question about perception on the size of babies at birth provided the only available measurement of a child’s health status at the time of delivery, as information about the exact weight of a child at birth was not available in most households. The question was prone to recall bias and measurement error. Further studies that collect the true actual size of the babies at birth are recommended.

Finally, there were other factors that were not captured by the questionnaire, for instance: the child rearing practice by mothers, household environment, and familial genetic problems. All these limitations were difficult to rectify through a single quantitative survey, warranting future studies to unpack these complexities.

## Conclusion and policy implications

In conclusion, this research found that childhood stunting was significantly associated with the following risk factors: poor economic status of a household, small size of a child at the time of delivery, and residential location in Bangkok. The study also suggested that prolonged breastfeeding beyond 12 months when in combination with poor economic status of a household potentiated the risk of stunting. Household wealth was protective factor against stunting in children who were breastfed for both short and prolonged periods of breastfeeding. Children living in the least well-off households were more prone to experience stunting than others. This study points to target interventions to identify and support the poorest households, using multi-sectoral actions such as poverty reduction, ensuring food security, health education and the empowerment of women. As long as household poverty remains an impediment, health education and supplementary food provision alone will not be sufficient to radically tackle the malnutrition of children.

## Abbreviations

95% CI: 95% Confidence interval
CI: Confidence interval
cm: Centimeter
EA: Enumeration area
HH: Household
kg: Kilogram
MCH: Maternal and child health
MDGs: Millennium Development Goals
MICS: Multiple Indicators Cluster Survey
OR: Odds ratio
SDGs: Sustainable Development Goals
SE: Standard error
UN: United Nations
WHO: World Health Organization
